# Residents' perspectives on the final year of medical school

**DOI:** 10.5116/ijme.5019.6b01

**Published:** 2012-08-07

**Authors:** Bridget Obrien, Brian Niehaus, Arianne Teherani, John Q. Young

**Affiliations:** 1Department of Medicine and Office of Medical Education, School of Medicine, University of California, San Francisco, CA, USA; 2 Department of Psychiatry, School of Medicine, University of California, San Francisco, CA, USA

**Keywords:** Undergraduate medical education, transition, personal and professional development

## Abstract

**Objectives:**

To characterize junior residents’ perspectives on the purpose, value, and potential improvement of the final year of medical school.

**Methods:**

Eighteen interviews were conducted with junior residents who graduated from nine different medical schools and who were in internal medicine, surgery, and psychiatry programs at one institution in the United States. Interview transcripts were coded and analyzed inductively for themes.

**Results:**

Participants’ descriptions of the purpose of their recently completed final year of medical school contained three primary themes: residency-related purposes, interest- or need-based purposes, and transitional purposes. Participants commented on the most valued aspects of the final year. Themes included opportunities to: prepare for residency; assume a higher level of responsibility in patient care; pursue experiences of interest that added breadth of knowledge, skills and perspective; develop and/or clarify career plans; and enjoy a period of respite. Suggestions for improvement included enhancing the learning value of clinical electives, augmenting specific curricular content, and making the final year more purposeful and better aligned with career goals.

**Conclusions:**

The final year of medical school is a critical part of medical education for most learners, but careful attention is needed to ensure that the year is developmentally robust. Medical educators can facilitate this by creating structures to help students define personal and professional goals, identify opportunities to work toward these goals, and monitor progress so that the value of the final year is optimized and not exclusively focused on residency preparation.

## Introduction

Medical schools and residency programs in the United States and abroad are redesigning medical education toward a competency-based, developmental continuum.^1^-^3^ This shift may allow movement away from a time-based model of education toward an outcomes-based model in which students would have the option to complete training early or pursue elective training after demonstrating competence in required areas.^4^ With this shift in mind, the purpose of the fourth, or final year, of medical school comes under scrutiny. Currently the fourth year in US and Canadian medical schools provides time for the arduous “audition” - the application and interview process associated with residency placement - as well as time for elective coursework and advanced clinical responsibilities in sub-internship (Sub-I) roles in one or two specialties.^5^ However, some have asked whether the year adds sufficient value to student learning and/or professional development to justify the costs.^6^ Arguments for shortening, eliminating, or reforming ensue.These debates reflect at least two perspectives on the purpose of the final year. The instrumental purpose focuses on the goal of matching to the residency program of choice and engaging in specialty-specific residency preparation. The holistic purpose focuses on opportunities to round out one’s medical education, including time for personal and professional development through a variety of clinical and non-clinical experiences. These dual purposes often play out in discussions around the relative proportion of required versus elective coursework in the final year and general versus specialized preparation for residency.^7^,^8^New visions of medical education suggest that students in their final year of medical school ought to demonstrate competence on standardized outcomes, pursue personal learning goals through individualized processes, progress toward proficiency in an area of interest (e.g. public policy, translational research, global health, medical education) and continue professional formation.^9^ A recent review of the literature on the fourth year highlighted gaps in the information available to guide change toward this new vision.^7^ The perspective of junior residents, as key informants about the transition from medical school to residency, can fill one of the gaps in the literature and provide insights that educators need to enact meaningful reforms. Junior residents’ perspectives can complement those of program directors and undergraduate medical educators that are already well documented in the literature.^10^,^11^Toward this end, we formulated the research question: what are junior residents’ perspectives on the purposes, perceived value, and suggestions for improvement of the final year of medical school? We then discuss these perspectives in relation to the literature and a synthetic set of principles to guide changes to the final year.

## Methods

### Approach

We conducted a qualitative study using an inductive approach to identify themes.^12^,^13^ A qualitative inductive approach was deemed appropriate to explore resident perspectives in depth with minimal a priori framing of the topic. This study was approved by the Institutional Review Board of the University of California, San Francisco (UCSF).

### Participants and setting

We used a purposeful sampling strategy. We selected early second-year residents because they were at a stage in their education where they could reflect on the final year of medical school, the first year of residency training, and the transition between the two. We selected three specialties (internal medicine, psychiatry, and surgery) to provide a range of procedurally and non-procedurally oriented perspectives. We purposefully sampled a combination of graduates from our own institution as well as from other institutions to provide insight to a variety of fourth year curricula. Beginning in August 2009, we invited 53 second-year residents in internal medicine (n=28), surgery (n=14), and psychiatry (n=11) to participate. At the beginning of each interview participants received an information sheet describing the risks and benefits of participation in a research study and the voluntary nature of participation in research. Names and identifying information were removed from all interview transcripts to protect the anonymity of participants during data analysis.

### Instruments

We created a semi-structured interview guide to address our research question. The semi-structured guide allowed us to ask a focused set of questions of each resident, while also allowing space for the interviewer to probe for greater depth on topics that uniquely emerged in individual interviews. To refine our interview questions we used the following sources: relevant literature, local evaluation data, focus group themes from final year students, and critical feedback from members of our institution’s fourth year curricular committee. Example questions include:

- “How did you decide which rotations and experiences to include in your 4^th^ year schedule?”
- “What do you think was the purpose of 4^th^ year for you?”
- “From your school’s perspective what did you perceive as the purpose of 4^th^ year?”
- “If you were running the 4th year curriculum, what do you think the goals of fourth year should be?”
- “Are there ways in which you feel 4^th^ year contributed to your professional development? If so, please explain. If not, why not.”

### Procedures

One author (BN) emailed invitations with up to 3 reminders to all residents, from September to November, 2009. This author (BN) conducted all interviews in person at the training site of each resident. Each interview was between 10 and 25 minutes long. Interviews were recorded and transcribed. The interviewer met with the other authors multiple times while interviews were occurring to debrief, review the interview guide and make minor modifications, and discuss emerging ideas and themes. We ended data collection in December 2009 when we exhausted the pool of volunteers.

### Analysis

We analyzed the interview transcripts thematically.^13^ Three authors (BCO, BN & JQY) independently reviewed three transcripts and proposed a list of coding categories. We compared, discussed, and combined our lists into a single coding scheme, then applied the coding scheme to two additional transcripts and identified additional categories needed. Again, we met, discussed and finalized our coding scheme. Two authors coded each transcript (BCO functioned as the primary coder, BN and JQY functioned as secondary coders). This approach allowed us to bring multiple perspectives to bear on the transcripts (medical education researcher (BCO), recent medical school graduate (BN), and clinician-educator (JQY)), to discuss different interpretations, and to reach a shared understanding of meaning. We reviewed coded passages to identify and connect overarching themes.

## Results

Eighteen residents volunteered for interviews: nine from internal medicine, four from surgery, and five from psychiatry. These residents graduated from nine different medical schools; half from the University of California, San Francisco. Twelve participants were female. Most residents said they had decided what specialty they would pursue by the time they started their final year (72%). Many of the residents took some time off between undergraduate education and medical school (39%) and/or during medical school (33%). Half received formal mentoring during fourth year (Table 1).

**Table 1:** Characteristics of PGY 2 Residents interviewed, by graduation from UCSF, timing of specialty choice, time off, and mentoring (N=18)

Variable	IM (n=9)	SU (n=4)	PSY (n=5)	Total (N=18)
Female	6	3	3	12
Graduated from UCSF	6	2	1	9
Deciding in 4^th^ Year	1	2	2	5
Time off prior to medical school	3	1	3	7
Time off during medical school	4	0	2	6
Received formal mentoring	3	3	3	9

We organized our results into three major sections aligned with our research question: purpose of the final year, perceived value of the final year, and suggestions for improvement.

### Purpose of the final year

While many residents conveyed some uncertainty about the purpose of fourth year (n=8), the three most prominent themes were residency-related purposes, interest- or need-based purposes, and transitional purposes. Residency-related purposes included completing residency applications and interviews, acquiring application-strengthening experiences, and/or preparing for internship through consolidation of specialty-specific skills and intern-like roles. Purposes such as pursuing career interests (research, teaching, global health) and filling perceived gaps in experience characterized interest or need-based purposes. Transitional purposes consisted of respite before internship. Several residents highlighted the value of time in fourth year to “decompress,” “take a break” between two very taxing years, “relax” and “enjoy life” (n=7). These purposes need not be mutually exclusive, as one resident explained:

*“It [fourth year] can still be kind of challenging and rewarding while at the same time providing a break from third year. I think all three of those things between choosing a specialty, continuing to work on things and also providing somewhat of a break*.” [PSY3]

In many cases, the perceived purpose of the final year was colored by whether or not the resident had chosen a specialty (n=15). Four residents described the primary purpose of the final year as choosing a specialty, while three who knew their specialty choice at the start questioned whether fourth year had much purpose or added value to their education.

*“I think in the current system the fourth year is only necessary for people who don’t know what they want to do with their life. Otherwise I think if you know you could potentially start to focus in towards the end of your third year with additional sub-I’s and be done with medical school in three years… maybe if it was something that could somehow be optional … or make it more rigorous and make it actually something worthwhile, because people are paying tuition.”* [IM4]

### Perceived value of the final year

#### Planning the final year

Residents tended to describe the final year of medical school in three phases organized around residency selection. These phases influenced scheduling choices and were perceived as more or less valuable for different reasons. The first phase was preparation for residency applications and interviews. Most residents recalled planning their schedule strategically for residency applications and interviews; many “frontloaded” their schedule, meaning they tried to do their sub-internships (sub-I’s) early so they could obtain grades, letters of recommendation, and desired experiences (n=12). Some residents who had taken a year off between third and fourth year of medical school strategically chose to do a few rotations to “warm-up” before beginning their sub-I’s (n=3). Five residents recalled using early fourth year experiences to confirm their future specialty.

*“During fourth year everyone thinks about when to do the sub-I for the field you want to apply to. I wanted to do a lot of the clinical stuff in the very beginning of my fourth year to buff up my application, kind of really making sure I want to go into the field that I want to go into.”* [IM9]

Thus, much of the value in this phase came from contributions to specialty choice and strengthening one’s residency application.The second phase was the interview season, which residents described as requiring significant time, effort, and flexibility. A few scheduled research to accommodate their interview plans (n=2); others described frustration with electives that had strict attendance policies (n=2). This phase was not explicitly identified as valuable and seemed generally accepted as standard fare for the transition to residency.The third phase occurred after application and interview season and was much less intense. Many residents remembered taking a more relaxed approach to the rest of their fourth year (n=10). Most chose electives that matched an area of interest or provided broad exposure to medicine, often in areas they expected not to have an opportunity to experience in depth or to do again.

*“My main objective of fourth year was completed by January and at that time it was based on interest, things I thought I would never get to experience again, that I thought would be fun and educational, and the last two months I just completely took off.”* [SU3]

Several used the time for research or other scholarly projects (n=5) and to travel and experience other health care systems (n=3). In retrospect, most residents valued the time and flexibility for these pursuits.

*“I took time to do a project that was completely removed from anything clinical. That space, the freedom they give you in your fourth year … is very, very, very valuable*.”[IM9]

#### Sub-Internships (Sub-I’s)

Residents described different sub-I requirements and advice depending on the medical school they attended and the specialty they selected. For example, some schools required a sub-I in internal medicine while others allowed students to choose any specialty for sub-I’s.Nearly all residents described the sub-I experience as valuable (n=17). The most frequently cited reasons were the level of responsibility and exposure to the role of an intern.

*“The internal medicine sub-I is one of the very few places… that you actually have the responsibilities of a sub-intern, carrying your own patients, reporting to the resident and not the intern, working the hours of an intern where nothing is really padded for you … I think that really gives you experience that you need for residency.”* [PSY1]

*“I got to the point where I thought, towards the end of my sub-I, that, yeah, I could be an intern. It’s not as scary any more. So I think it did give me a mental preparation, as well as the skill-set and knowledge base, to feel that I could become an intern.”* [IM7]

Particularly valuable aspects included taking call, carrying a panel of patients, and writing orders. For career choice, the sub-I allowed students to make sure they liked the patient population, types of diseases, critical thinking, and therapies common to their chosen specialty. The sub-I provided important experiences to draw upon during residency interviews.

#### Clinical and non-clinical electives

In general, residents felt most electives were less valuable for residency preparation than sub-I’s.

*“I would say everything else [besides sub-I and ICU rotation] really turned out to be of interest at the time, but didn’t really add any level of preparation for internship.”* [IM4]

The electives identified as valuable had several common characteristics. They related to the specialty of choice and focused on broadly applicable knowledge or skills that seemed useful for physicians in any specialty (e.g. radiology, dermatology). Also, these electives had good mentors or teachers.

#### Personal / professional development

Most residents described ways in which fourth year contributed to their career development. This included specialty decisions, residency selection, whether or not research was important for future work, and exploration of other areas of interest such as global health, public health or medical education (n=12).

*“It was a chance to give me a little bit of variety but at the same time explore things that I knew I wanted to do just to see where my career could go.”* [PSY4]

Several residents saw a reflective component to the final year of medical school (n=6), both in a formal, structured way through courses as well as informally, because of the flexibility and numerous choices that needed to be made.

*“For me the flexibility forced me to do some self-evaluation about what would be more useful to me.”* [PSY2]

The final year also permitted confidence-building and transitioning from student to physician. A few residents noted that in the fourth year they began to appreciate how much they had learned in third year, experience more confidence in their knowledge and skills, and feel ‘ready’ to begin internship (n=4).

*“For example a sub-I, where you have patient care responsibilities, are the opportunities where your true professional character grows, where you start to realize the bigger picture. “I’m now transitioning away from ‘this is about me and my learning’ to ‘I’m actually delivering patient care to someone in a very vulnerable moment in their lives.’” It requires you to bring your “A-game” every day, which is a very big part of professionalism, and that only happens with true workplace learning and when you have that responsibility given to you.”* [IM4]

Only two out of the eighteen residents questioned whether the final year contributed much to their overall personal and professional development.

#### Mentoring / Advice

Half the residents recalled receiving formal mentoring or advising about residency and career planning from faculty in the final year. However, many reported that students at the end of their final year or junior residents provided the most valuable advice (n=8). Because these learners had recently experienced the process, they could help students choose sub-I’s and electives and provide practical advice about applications and interviews. A few residents suggested adding formal opportunities for students to get advice from junior residents (n=4).

*“If you could get residents to volunteer, they’re probably the best people to walk you through the process – because it is a process*.” [SU4]

A few residents felt they did not need a formal mentoring process during the final year of medical school, particularly if their own career goals were clear (n=4). They felt comfortable seeking advice as needed from residents and faculty. However, three residents who did not receive mentoring felt a formal mentoring program might have been beneficial. A few residents felt that more guidance through the interview and application process would have been helpful (n=3) and two residents, who recalled making their specialty or career decisions in fourth year, expressed some desire for better support during the decision process.

### Suggestions for improvement

Residents had suggestions for improving the final year of medical school. They highlighted specific content and experiences for inclusion, based on utility, relevance and/or gaps experienced during internship, such as: evidence-based medicine, critical care, procedures labs relevant to chosen specialty, EKG, and transition to internship. In addition to curricular content, they suggested: 1) better preparation for internship; and 2) clearer and more specific educational and professional goals for the fourth year as a whole and for the individual student.Most residents described the transition to internship as very challenging and filled with stress and anxiety (n=14). Although a few residents identified specific areas of knowledge or skill where they felt unprepared, most were less concerned about these areas because the gaps were filled within the first few months of internship.

*“To be honest, yes, more sub I’s, more procedures will probably better prepare you for internship, but internship itself is a very steep learning curve and sometime along the way you’ll probably learn the stuff that you need to learn, so I don’t know if it’s that much better if you learn it two or three months ahead of time versus on the job*.”[IM3]

The greater challenges were the increased workload and the level of responsibility.

*“You’re admitting twice as many patients. So the volume – I wasn’t expecting that at the beginning and the complexity of the patients.”* [IM3]

*“The most overwhelming thing is how to organize your time and your prioritization of what you do first in the day and later in the day and things like that.”* [SU2]

*“The hardest thing is just being used to the title and the responsibility that came with it and doing simple things like writing orders.”* [Psych 5]

For these challenges, the most common suggestion was to provide more practice in a role with progressive responsibility including: carrying a pager, responding to and prioritizing calls from nurses that required management decisions about patients, and managing workload.

Most residents supported the flexible structure of the final year and felt this should be preserved. However, some felt the level of rigor could be improved, particularly by helping students define clearer goals and then making appropriate curricular plans. Most saw problems with adding more required courses, given the variable interests, needs and specialty-focus of students in the final year.

*“I hate to say this because fourth year was such a fun year, but it may be beneficial to require a few more sub I’s, require a little bit more dabbling in things that may not be what you plan on doing for a career because third year only provides a limited level of expertise … I can see that perspective now, that I’m so developed in medicine and not my other skills. That was my last chance, and I sort of regret the fact that I had the opportunity to just blow off a lot of it here where I could have learned a lot, and that may have been my own failing, but I have a feeling that a lot of people did that.”* [IM4]

## Discussion

From the perspective of second-year residents, the final year of medical school in the United States serves both instrumental and holistic purposes by providing opportunities to: prepare and strengthen one’s residency application; assume a higher level of responsibility in patient care; begin the transition from student to physician (intern); pursue experiences of interest and/or that add breadth of knowledge, skills and perspective; develop and/or clarify career plans; and enjoy a period of respite. Additionally, several residents supported defined competencies and milestones for the final year; enhanced mentoring or advising and other strategies to help students develop a curricular plan organized around individual learning goals; and opportunities for synthesis, reflection, and consolidation of knowledge and skills to facilitate the transition to internship.Residents’ descriptions of instrumental, or residency-related, purposes highlight the pivotal role that residency selection plays during the final year of medical school. For decades, educators have raised concerns about “pre-residency syndrome.”^14^ Similarly, our findings suggest that residency is commonly perceived as the primary goal or “outcome” and that application, interviewing, and selection processes dominate much of the planning and choices that occur during the final year, including both the type and sequence of rotations. Unless the residency match process changes, its prominence in the final year will remain strong and educators will need to ensure that robust educational structures and supports are in place to promote the other important purposes.Our synthesis of residents’ perspectives and existing literature highlight three key elements of a framework for standardization and individualization in the final year of medical school: specification of core competencies and milestones for students to achieve during the final year and by the end of medical school, individualization of learning plans and processes, and sufficient support and accountability for required and personal outcomes.

### Specification of competencies and milestones

Establishing and aligning competencies and milestones that map the critical outcomes to be achieved over the course of medical school and graduate medical education would provide clearer goals for students in their final year of medical school. Currently, as evident in our interviews, students must make a giant leap from medical school to internship, suddenly carrying a panel of patients several times larger than they carried as a sub-I, writing orders, and receiving and responding to a broad range of clinical questions and emergencies (e.g., pages). In a competency-based system with developmental milestones, the learning trajectory should be more evenly progressive and the transitions should be a manageable step in this progression. These competencies could be linked to the existing accreditation for graduate medical education (ACGME) competencies, but with more specifics for the final year of medical school and the transition to residency.^15^ Defining critical competencies also provides an anchoring point for the final year such that unmet competencies become a primary focus to ensure graduation and readiness for internship and residency. After demonstrating competencies, students can focus on other personal and school-defined goals. For example, schools may specify goals such as completion of a scholarly project or acquisition of expertise in a defined area of interest. Several medical schools have already initiated this process.^1^-^3^,^16^

### Individualization of learning plans and processes

Although some residents in our study had a clear sense of what they wanted to accomplish during their final year beyond selection of and match to their residency of choice, most did not and none described developing a formal learning plan for their final year. However, several suggested that some sort of plan and accountability could have helped focus their experiences. One model is the individual learning plan (ILP) used in pediatric residencies to facilitate reflection on career goals, self-assessment of areas of strength and weakness, generation of goals, development of plans or strategies to achieve the goals, and assessment of progress toward goals.^17^ Tracking progress toward goals was associated with greater resident progress toward goals.^18^ Although there is little description of ILPs in medical school, they are meant to be adaptable for learners at all levels.^17^ Implementing ILPs in the third year and sustaining them into the fourth year may allow for guided individualized learning and more purpose-driven elective plans.

### Support and accountability structures

The third component includes all the supporting resources, structures, and systems of accountability needed to facilitate students’ achievement of required outcomes and personal goals. An effective system for guidance, mentoring and advising is a key supporting structure in this framework and one that coincides with ILPs. Most residents felt at least some level of guidance was needed in their final year of medical school, typically on practical issues such as designing a schedule for the year or applying and interviewing for residency and/or on professional development issues such as recommendations for exploring areas of interest and pursuing career goals. For practical issues, residents’ suggested adding a reliable system of near-peer mentoring. Similar to near-peer teaching,^19^ guidance from interns and residents may be better aligned with the needs and priorities of fourth year students than guidance from faculty members, at least for information about recommended courses, experiences to prepare for internship, and residency application. For longer-term professional development, students typically need mentoring and advice from faculty members. Ideally, these relationships should begin early in medical school and be connected to a structured program.^20^ The Colleges Program at UCLA is one example of a successful mentoring program and curriculum that supports students in practicalities like scheduling electives and preparing residency applications as well as in career and professional development.^5^,^21^The success of both competency-based requirements and individualized learning plans also requires robust courses and experiences that can help students achieve required competencies and progress toward self-identified instrumental and developmental goals. Nearly all residents described sub-Is as a valuable experience to prepare them for internship, largely because they had a more significant role in patient management and greater responsibility for patient care. Efforts have been underway in the last decade to standardize the curriculum of medicine sub-Is to further improve quality and consistency.^22^-^24^ The quality of the non-sub-I clinical electives could be enhanced by incorporating many of the features of sub-I’s such as actively engaging students in patient care or giving them intern-like responsibility for patients.In addition to sub-I’s and clinical electives, residents suggested providing structured time during the final year for students to review key concepts and skills, participate in focused preparation for internship, and reflect upon and synthesize their medical school experience. Several of the specific content suggestions paralleled essential fourth year competencies identified by program directors (e.g. advanced clinical reasoning, near intern-level independence, self-reflection and improvement, and effective use of evidence-based medicine).^10^A variety of structured courses that have demonstrated effectiveness in terms of student and resident satisfaction and perceived preparation for internship are described in the literature.^25^-^31^ By adding structure and accountability while preserving substantial elective time for customization, these interventions are consistent with the vision of a fourth year that provides both standardization and individualization.Experiences beyond clinical medicine, in areas such as research, global health, policy, and medical education, were recognized by residents as an important part of their personal goals and professional development. Yet, these are often individualized experiences, undertaken opportunistically with relatively little formal structure or oversight, which can result in limited rigor and uncertain outcomes. The development of ILPs and robust mentoring / advising systems will likely help direct students toward higher quality experiences.Finally, while residents embraced structures for support and accountability, they also frequently mentioned the importance of having time for a break between two very intense years of training. Few residents regretted taking vacation or personal time or even just a more “laid back” set of courses, noting that it provided a needed break between the stress of third year and the beginning of residency. Studies of medical student burnout further suggest that time to fulfil holistic purposes that have little to do with preparation for residency may be beneficial, particularly for students who are less resilient to burnout.^32^Our study has limitations. While it adds the perspective of junior residents to those of other stakeholders, our sample only represents residents from nine medical schools, three specialties, and three top programs at a single institution who volunteered to be interviewed. One-third of the residents in our sample took a year off during medical school, which is consistent with the pattern for students at our institution but may not be representative of all medical schools. Further work is needed to determine whether the findings from our study resonate with the views of residents from a wider range of specialties, medical schools, and residency programs. Recall of specific details of the final year of medical school may be imperfect since more than a year had passed since graduation. However, we intentionally selected for the time lag to allow reflection on fourth year after completing internship.Our study has implications for future research in medical education around the transition from student-in-training to physician-in-practice. Much remains unknown about the optimal balance between formal requirements (standardization of content and outcomes) and elective experiences (individualization of learning experiences) to maximize personal and professional development in the final year of training. International comparisons on this topic may offer important insights given the variability in the length of training and the types of career decisions confronting learners at this transition point.

## Conclusion

While debates about the purpose, value, and optimal design of the fourth, or final, year of medical school will likely continue in the current era of competency-based education, our study suggests that a developmentally robust final year is a critical part of medical education for many learners. Although the pressures associated with residency applications, interviews and match to residency programs cannot be ignored, other important purposes of the final year warrant attention. Standardization of competencies and outcomes needed to graduate and enter residency may provide a road map and baseline goals to guide students through their final year. Additionally, opportunities for students to define personal goals and achieve them through individualized learning and personal/professional development can help students focus on interests beyond baseline requirements. Finally, a strong and comprehensive support system is an important enabling factor for standardization and individualization. With these features, the final year of medical school can play a critical role in the education of many future physicians.

## 

### Conflict of Interest

The authors declare that they have no conflict of interest.
